# Asthenopia Due to Disorders of the Stomach

**Published:** 1910-01

**Authors:** H. L. Hilgartner

**Affiliations:** Austin, Texas


For Texas Medical Journal.

### Asthenopia Due to Disorders of the Stomach.

BY H. L. HILGARTNER, M. D., AUSTIN, TEXAS.

Diseases of the eye often possess important significance in relation to the diagnosis and correct understanding of diseases of other organs; and as both the physician and the ophthalmologist should comprehend the mutual relations of such affections, both in diagnosis and treatment, it is necessary to consider the relations of the eye and its diseases to diseases of the rest of the body. While these relations are usually more or less referred to in text-books, they are not set forth with such particularity as is often desirable. Frequently eye symptoms apparently insignificant lead to far-reaching deductions concerning the primary and originating affection. Example: Ecchymosis under conjunctiva, albuminuric retinitis, etc.

In this paper I will only refer to one class of cases included under the heading, eye symptoms caused by stomach diseases.

Abnormal digestive processes are very frequently causes of general anemia and hydraemia with their sequelae, feeble accommodation, weakness of the external ocular muscles (insufficiency).

In severe cases hemorrhages also take place into the retina, rarely into the vitreous body. Neuralgic processes (and certain asthenic uveal diseases, such as iritis serosa) develop in individuals with impaired nutrition.

Frequently patients with headache and asthenopia consult oculists, thinking that glasses will correct neuralgia or painful vision. Upon very careful examination of the eye and its appendages, no defect—at least not sufficient to account for the trouble—can be detected. Then it is the duty of every oculist to look beyond the eye for the cause of the trouble. In many such cases, I had my attention directed to the stomach, and accordingly advised the proper examination. In most instances the examination (in my experience) has revealed an excessive acidity with excessive secretion. In quite a number of such cases, I can report entire relief from all eye symptoms, with proper local and constitutional treatment directed at the stomach, and no treatment for the eye. It is a well known fact that a specialist is prone to attribute all "the ills that flesh is heir to" to an affection of some organ which belongs to his particular specialty. It is, therefore, important to guard against this weakness and to view patients with the greatest possible broadness. I do not wish to deprecate necessary eye treatment in this particular class of patients: if we find sufficient error of refraction, the proper lens should be ordered; if muscular insufficiency is found, suggest such treatment as seems best suited for the case.

In this connection I wish to say that I have noticed stomach diseases to have caused the most misleading muscular unbalance. One examination would show insufficiency of the ext. recti, while the next test would indicate weakness of the int. recti. I think it safe to state that whenever this vacillating muscular trouble is noticed, we can look for some cause elsewhere than in the eye itself. We find in the secondary ocular affections that we have to deal with toxic symptoms, in the broadest sense of the term, from the most acute to the most chronic. The differences are due mainly to the fact that in one case the virus is introduced into the body; in another it is produced in the body, either with or without the aid of living elements. Improper articles of diet give rise to changes in the stomach and intestines, and, secondarily, to changed chemical conditions of nutrition in the entire organism. In these auto-intoxications we also find that a certain virus attacks certain organs more than others. These organs are either the site of the greatest accumulation of the virus, or their constituents are especially sensitive to the virus.

